# Failure to Recognize Nontuberculous Mycobacteria Leads to Misdiagnosis of Chronic Pulmonary Tuberculosis

**DOI:** 10.1371/journal.pone.0036902

**Published:** 2012-05-16

**Authors:** Mamoudou Maiga, Sophia Siddiqui, Souleymane Diallo, Bassirou Diarra, Brehima Traoré, Yvonne R. Shea, Adrian M. Zelazny, Bindongo P. P. Dembele, Drissa Goita, Hamadoun Kassambara, Abdulrahman S. Hammond, Michael A. Polis, Anatole Tounkara

**Affiliations:** 1 Project SEREFO-NIAID/University of Bamako Research Collaboration on HIV/TB, Bamako, Mali; 2 CCRB, Division of Clinical Research, National Institute of Allergy and Infectious Diseases, Bethesda, Maryland, United States of America; 3 Microbiology Service, Department of Laboratory Medicine, Clinical Center, National Institutes of Health, Bethesda, Maryland, United States of America; Universidad Nacional de La Plata, United States of America

## Abstract

**Background:**

Nontuberculous mycobacterial (NTM) infections cause morbidity worldwide. They are difficult to diagnose in resource-limited regions, and most patients receive empiric treatment for tuberculosis (TB). Our objective here is to evaluate the potential impact of NTM diseases among patients treated presumptively for tuberculosis in Mali.

**Methods:**

We re-evaluated sputum specimens among patients newly diagnosed with TB (naïve) and those previously treated for TB disease (chronic cases). Sputum microscopy, culture and *Mycobacterium tuberculosis* drug susceptibility testing were performed. Identification of strains was performed using molecular probes or sequencing of *secA1* and/or 16S rRNA genes.

**Results:**

Of 142 patients enrolled, 61 (43%) were clinically classified as chronic cases and 17 (12%) were infected with NTM. Eleven of the 142 (8%) patients had NTM disease alone (8 *M. avium*, 2 *M. simiae* and 1 *M. palustre*). All these 11 were from the chronic TB group, comprising 11/61 (18%) of that group and all were identified as candidates for second line treatment. The remaining 6/17 (35.30%) NTM infected patients had coinfection with *M. tuberculosis* and all 6 were from the TB treatment naïve group. These 6 were candidates for the standard first line treatment regimen of TB. *M. avium* was identified in 11 of the 142 (8%) patients, only 3/11 (27.27%) of whom were HIV positive.

**Conclusions:**

NTM infections should be considered a cause of morbidity in TB endemic environments especially when managing chronic TB cases to limit morbidity and provide appropriate treatment.

## Introduction

Nontuberculous mycobacterial (NTM) infections cause morbidity worldwide [1]. Currently more than 125 different types of NTM exist in the environment. Those implicated in human disease can produce nonspecific symptoms [2], with clinical manifestations ranging from no symptoms or signs to destructive or even fatal disease. When symptoms and signs occur, they are often indistinguishable clinically and radiographically from those caused by *Mycobacterium tuberculosis* [3]. However, the pattern of resistance and treatment outcomes for NTM disease can be significantly different from TB, resulting in different implications for public health [4].

Though widely recognized for causing symptomatic disease in developed countries, the role of NTM in pulmonary or systemic disease in developing countries is not well described. This is largely attributable to lack of diagnostic facilities to culture and identify mycobacteria. Since TB is usually endemic and life threatening in these areas, patients are presumptively treated for TB.

At present, there is no specific information regarding the role of NTM in causing infection or disease in Mali, where this study was conducted. Patients are treated based on sputum smear exams using standard first and second line TB therapy depending on clinical criteria in conjunction with World Health Organization (WHO) guidelines [5]. As NTM are often resistant to first-line anti-TB medication, presumably many of these cases would be considered treatment failures, and subsequently treated for multi-drug resistant (MDR) disease. In this study, we evaluated a cross section of either naïve or chronic TB patients for NTM infection alone or a coinfection with *Mycobacterium tuberculosis (M.tb)*. For these patients, we determined species of NTM, susceptibility to TB drugs and HIV status.

Since NTM are ubiquitous in the environment, infection and disease due to NTM were differentiated by clinical evidence based on the American Thoracic Society (ATS) criteria [6].

## Methods

### Ethics Statement

Patients were recruited on protocols approved by the Institutional Review Board of the National Institutes for Health (NIH), USA and the University of Bamako's Ethic Committee in Mali. The protocols were conducted in compliance with all international standards applicable to human studies, including written informed consent obtained from all the volunteers.

### Setting/Participants

The study took place at the HIV/TB Research Project, SEREFO, of the University of Bamako, in Mali. The participants of this study represent a cross section of TB treatment-naïve and chronic TB patients identified in the study clinics by the study clinicians from March 1, 2004 to November 30, 2009. Patients were recruited consecutively on protocols. The city of Bamako is administratively divided into six municipalities each with a local referral health center capable of diagnosing and treating TB patients. These centers refer TB treatment failure cases to the main TB clinic of the city at Point-G hospital.

Naïve patients were recruited directly from one of the 6 local centers. Patients who had sputum smear positive disease were referred to the study team by their physician at the referral center. All chronic patients presenting to the TB Clinic at Point-G Hospital (the only center specialized in the treatment of chronic TB and MDR-TB in the country) during the study period were referred to the research team.

### Study Design

This was a clinic-based, cohort design, in which all patients seen for presumptive TB (at least 1 sputum smear positive at the referral center and no history of TB treatment, (naïve cases) or for TB treatment failure (with at least 2 sputum smears positives after 9–13 months of treatment (chronic cases) during the study period were offered enrollment. Standardized interviews accompanied by physical examination were conducted and 3 consecutive early morning sputa were collected on 3 consecutive days. The three consecutives sputa (in three days) for the study purposes were collected before commencing treatment for naive patients or retreatment for chronic patients. The sputa were self collected each morning by the patients and then immediately transported to the study clinic. The sputum collection was not repeated during patients' treatment or retreatment.

Blood for HIV testing was obtained from each volunteer. The variables collected during the physical exam for each participant were: age, sex, occupation, symptoms and the medical history including previous treatments.

### TB Treatment Regimen

Patients naive to treatment received the standard TB regimen from their physicians (Table 1). Those with positive sputum smears after 5 months of the standard regimen received the retreatment regimen while those with sputum smears positive after 13 months (5 months of standard regimen and 8 months of re-treatment regimen) received the second line treatment (Table 1). Those completing the retreatment regimen, receiving or commencing the second line regimen at enrollment are referred to, as chronic TB patients.

**Table 1:** *Mycobacterial species* isolated, demographic characteristics, symptoms and treatment assigned to each patient.

Patient	Age	Gender	HIV Status	Occupation	Symptoms	AFB Smear	AFB Culture	NTM Isolated	Co-infection	Molecular ID tools used	Treatment at time of enrollment$
**1**	31	Male	Neg	Driver	Weight loss, fever, headache	No AFB	Pos	*M. avium*	*M.tb*	Probe	Standard regimen*
**2**	53	Male	Neg	Health Tech.	Cough, weight loss, fever, chest pain	Many AFB	Pos	*M. avium*M.moriokaense@	*M.af*	Probe	Standard regimen
										Sequencing	
**3**	18	Male	Neg	Construction worker	Cough, fever, chest pain	Few AFB	Pos	*M. kubicae*	*M.af*	Sequencing	Standard regimen
**4**	24	Male	Neg	Tailor	Cough, weight loss, fever	Few AFB	Pos	M.kumamotonense@ *M. fortuitum*	*M.tb*	Sequencing	Standard regimen
										Sequencing	
**5**	36	Male	Pos	Driver	Cough, weight loss, chest pain, dyspnea, anorexia	Few AFB	Pos	*M. avium*	*M.tb*	Probe	Standard regimen
**6**	36	Male	Neg	Cleaner	Cough, fever, anorexia	Many AFB	Pos	*M. gordonae*	*M.af*	Probe	Standard regimen
**7**	51	Male	Neg	Farmer	Cough, fever, anorexia, dyspnea, chest pain	Few AFB	Pos	*M. avium*	None	Probe	Second line treatment
**8**	56	Male	Neg	Accounts manager	Dyspnea, epigastric pain	No AFB	Pos	*M. avium*	None	Probe	Second line treatment
**9**	31	Male	Pos	Tailor	Cough, weight loss, fever	Many AFB	Pos	*M. avium*	None	Probe	Second line treatment
**10**	35	Male	Neg	Farmer	Cough, fever, hemoptysis	Many AFB	Pos	*M. simiae*	None	Sequencing	Second line treatment
**11**	34	Male	Neg	Accountant	Wasting, cough, rales	Many AFB	Pos	*M. avium*	None	Probe	Second line treatment
**12**	46	Male	Pos	Tailor	Cough, weight loss, chest pain, Dyspnea, anorexia	Many AFB	Pos	*M. avium*	None	Probe	Second line treatment
**13**	65	Male	Neg	Teacher	Weight loss, conjunctival palor, wheezing	No AFB	Pos	*M. avium*	None	Probe	Second line treatment
**14**	50	Female	Neg	Housewife	Cough, fever, dypsnea, chest pain	Few AFB	Pos	*M. simiae*	None	Sequencing	Second line treatment
**15**	40	Male	Neg	Cattle breeder	Cough, Headache, anorexia	Many AFB	Pos	*M. palustre*	None	Sequencing	Retreatment regimen
**16**	73	Male	Neg	Cultivator	Cough, weight loss, fever	Many AFB	Pos	*M. avium*	None	Probe	Retreatment regimen
**17**	44	Male	Neg	Mechanic	Cough, fever	Few AFB	Pos	*M. avium*	None	Probe	Second line treatment

@ *Mycobacterium species* most closely related;

* Patients were naïve to TB treatment before they were enrolled in the study and received the TB standard regimen for their disease.

$ Based on National treatment guidelines for TB, the Standard regimen comprises 2 months of rifampin, isoniazid, pyrazinamide and ethambutol and 4 months of isoniazid and rifampin (2RHZE/4RH). Patients with sputum smears positive at month-5 of standard regimen, receive 1 month of rifampin, isoniazid, pyrazinamide, ethambutol and streptomycin followed by 2 months of rifampin, isoniazid, pyrazinamide, ethambutol and 5 months of rifampin, isoniazid and ethambutol (2RHZES/1RHZE/5RHE called re-treatment regimen). The second line treatment for chronic cases (patients with sputum smears positive after re-treatment regimen) comprises kanamycin, ofloxacin, ethionamide and pyrazinamide (3KOEtZ/18OetZ) for MDR disease. *M.af: Mycobacterium africanum*; *M.tb: Mycobacterium tuberculosis*; *Neg: negative*; *Pos: positive*; *AFB: acid-fast bacilli.*

### Microbiology Testing

All samples were processed at the BSL-3 facility in Bamako, Mali unless mentioned otherwise.

Sputum specimens were decontaminated by the N-acetyl-L-cystein/sodium hydroxide method. The sediment was used to perform sputum smears (fluorescent Auramine Rhodamine staining) and to inoculate cultures. Culture was performed using Mycobacteria Growth Indicator Tubes, (MGIT, BD Sparks, MD, USA) and selective 7H11 Middlebrook agar plate. The cultures were incubated in CO_2_ for 42 days before being reported as negative. When a possible NTM was isolated on the plate, the colonies were sub-cultured onto new plates and the old plate was re-incubated until day 42.

### Species Typing

Nucleic acid probes (AccuProbe, Gen-Probe, San Diego CA. USA) for *M. tuberculosis* complex (MTBC), *M. avium* complex (MAC), *M. intracellulare*, *M. gordonae* and *M. kansasii* were used for initial identification. Eight samples, not identified by AccuProbe were sequenced and identified as *M. kumamotonense*, *M. kubicae*, *M. morokaense*, *M. fortuitum*, *M. simiae* or *M. palustre*. DNA was amplified by PCR followed by sequencing of the *secA1* gene [7] and/or full sequencing of the 16S rRNA gene with “MicroSeq Full Gene 16S rRNA Bacterial Isolation Sequencing kit” (Applied Biosystems, Foster City, Calif.) according to the manufacturer's protocol. Both phenotypic and genotypic characteristics were taken into consideration when identifying the isolate. The macroscopic colonial morphologies were consistent with the sequencing identifications for all of the isolates. Sequencing was performed at the National Institutes of Health, Bethesda, Maryland.

To distinguish *M. tuberculosis* from *M. africanum* strains, spoligotyping was performed using a commercial kit (Isogen Life Science, De Meern, The Netherlands) [8]. Strains comparison was made with databases available from the New York City Department of Health (NYC) and SPOTCLUST (SpolDB4-based).

### HIV Testing

“Determine HIV-1/2” (Abbott, Tokyo, Japan), Genscreen and Western Blot kits (BioRad, Paris, France) were used to determine the HIV serological status of the volunteers.

### Drug susceptibility tests

Susceptibility testing of *M. tuberculosis* was performed using BACTEC-MGIT 960 SIRE Kits (Franklin Lakes, NJ, USA). All MDR-TB isolates were sent to National Jewish Hospital, Denver, Colorado for confirmation and to perform second line drug testing by agar proportion method [9].

### Statistical analysis

The chi-square test with GraphPad Prism version 5 (GraphPad Software, San Diego, CA, USA) was used for the statistical analyses. A p-value less than 0.05 was considered significant.

## Results

During the study period, a total of 142 patients were referred to the study investigators and all were enrolled. Eighty-one patients (57.04%) were naïve to treatment (naïve group) and were recruited from the referral centers; 61 (42.96%) had a history of treatment with TB standard regimen or re-treatment regimen and were recruited from the Point-G hospital. These 61 were being evaluated for or were receiving treatment of multidrug resistant (MDR) disease. The median age of the patients was 43 years (range 18–73 years) with 109 males and 33 females. Most of these patients lived in Bamako or in the vicinity. NTM were isolated from 17 out of the 142 patients (12%). NTM were less prevalent in patients with naïve TB (6 of 81 or 7.4%) as compared to those with chronic TB (11 of 61, or 18%) [p = 0.03].

Fourteen of the 17 NTM patients (82.35%) were sputum smears positive with auramine rhodamine staining performed in our laboratory. All 17 were culture positive for mycobacteria on at least 2 occasions (Table 2) and presented with clinical symptoms consistent with TB (Table 1). The most common symptoms in NTM infected patients were cough 13/17 cases (76.47%), fever 11/17 cases (64.7%) weight loss 8/17 cases (47.05%), chest pain 6/17 cases (35.29%), anorexia 5/17 cases (29.41%) and dyspnea 5/17 cases (29.41).

**Table 2:** Impact of NTM on TB sputum smears or cultures.

Patients	Sputum smears		Sputum cultures	
	*Pos	#Neg	*Pos	#Neg
NTM alone (NTM disease)	9	2	11	0
NTM and MTBC	5	1	6	0
MTBC alone	108	5	113	0

*NTM: Nontuberculous mycobacteria*; *MTBC: Mycobacterium tuberculosis* complex;

* *Pos: positive in at least two occasions*;

# *Neg: negative in three occasions*.

### Naïve Group

All 6 of the 81 (7.4%) volunteers in the naive group infected with NTM (including one patient with *M. gordonae*) were co-infected with MTBC organisms, with 4 cases of *M. tuberculosis* (sensu stricto) and 2 cases of *M. africanum*. In 2 of the 6 volunteers (33.33%), we identified 3 different mycobacterial species: the first one was infected by *M. avium* and *M. moriokaense* in addition to *M. africanum* and the second one had *M. fortuitum* and *M. kumamotonense* in addition to *M. tuberculosis* (Table 1). One other patient (16.67%) had a coinfection of *M. africanum* with *M. kubicae*. All 6 co-infected patients were receiving standard treatment for TB, but not for NTM infection (Table 1).

### Chronic Disease Group

None of the 11 patients in this group with NTM were co-infected with MTBC (Table 1). Eight of the 11(72.73%) had MAC infection, 2 (18.18%) had *M. simiae* and 1 (9.09%) was infected by *M. palustre*. All these 11 patients with NTM met the ATS 2007 revised criteria [6] for NTM disease (Table 1). To the best of our knowledge the clinical response to the diagnosis of an NTM infection was cessation of TB treatment with no treatment being provided for NTM disease except for one patient. Follow up data for this patient (Patient 9 in Table 1) who had MAC infection was available. He was treated for MAC disease but returned 8 months later with continued clinical symptoms. Samples obtained at that time showed a diagnosis of MDR-TB and *M. avium* was not isolated.

### Drug Susceptibility Testing

In the chronic disease group 22/61(36%) with presumed MDR-TB actually had MDR disease. Six (9.83%) patients had fully susceptible strains, 10 (16.40%) had resistance to only one antibiotic, 11 (18.03%) had NTM disease and 12 (19.67%) patients were culture negative. In the treatment naïve group, only two patients (2.46%) had disease with an MDR strain as the cause of their primary infection. MDR disease was more common in patients who had chronic disease (22 of 61, or 36.06%) as compared to patients who were treatment naïve cases (2 of 81 or 2.5%) [(Table 3), p<0.0001].

**Table 3:** Distribution of patients in the naïve and chronic treatment groups based on culture and susceptibility results.

	Culture Negative	MTBC* Pansensitive	MTBC with at least 1 drug resistant and not MDR-TB	MDR#	NTM@	Coinfection (MTBC+NTM)	Total
**Chronic Disease Group**	12	6	10	22	11	0	61
**Treatment Naïve Group**	0	69	4	2	0	6	81
**Total**	12	75	14	24	11	6	142

* *M. tuberculosis* complex,

# Resistant to isoniazid and rifampin,

@ Nontuberculous Mycobacteria.

### Microbiology Data

Sputum samples from all 81 patients in the naïve group (100%) and 53/61 (86.89%) patients in the chronic group were sputum smear positive for AFB. Nine of the 11 patients (81.81%) presenting with NTM alone, had AFB positive smears (Table 2). All 81 acute cases were culture positive while 12 out of the 61 chronic cases (20%) were culture negative (Table 3).

Among patients coinfected with more than one mycobacterium species, colonies were clearly differentiated by culture and confirmed by subsequent probe, sequencing or both. For Patient 4, *Mycobacterium sp.* most closely related to *M. kumamotonense* and *M. fortuitum* were isolated on 2 separate visits, possibly indicating that they were colonizers rather than pathogens in the setting of *M. tuberculosis* coinfection. Sequence-based identification of mycobacteria in our institution is routinely performed by partial sequencing of *secA*1. Full sequencing of 16S rRNA gene is performed when *secA1* sequencing does not yield a conclusive identification (usually because of the species are not in the database). For most isolates for which both gene targets were sequenced, the 16S rRNA sequence homology was 99.7–99.9% and the corresponding *sec*A1 sequences (with the exception of *M. kubicae*) were 99.4–100%. *M. kubicae* was not present in the *secA1* database and therefore could not be evaluated.

Based on sequence analysis using 16S rRNA gene, Patient 2 had a mycobacterial isolate 99.1% similar to *M. moriokaense* group and Patient 4 had a mycobacterial isolate 99.2% similar to *M. kumamotonense*. Results of *sec*A1 sequencing were not definitive for any of these two isolates. Because the percent similarity of these isolates with 16S rRNA gene sequences is relatively low, we identified them as *Mycobacterium* species most closely related to *M. moriokaense* and *kumamotonense* respectively.

Interpretation of sequencing results can be controversial as there are no clear cutoffs. However, it has been suggested that similarity >99.5% is acceptable [10].

MAC infection seen in 11/17 (64.70%) patients was the most prevalent in the NTM infection (Table 1 et 3).

### HIV Infection

Twenty-eight of the 142 patients (19.71%) were HIV infected. Twenty-five of the 28 (89.3%) were acute (naive) TB patients and 3 (10.7%) were chronic TB patients. This frequency of HIV in TB patients is very close to the Mali National TB program's report of 2009 regarding the prevalence of HIV co-infection among TB patients, which was estimated to be 18%.

Three of the 28 (10.71%) HIV patients (1 naive case and 2 chronic cases) were infected by NTM. All 3 were infected by MAC (Table 1). The patient from the naïve group was coinfected with *M. tuberculosis*. All were receiving treatment for TB disease only and none were receiving antiretroviral therapy at the time of TB diagnosis. In addition, NTM infections were not more common in the HIV patients (3/28 or 10.71%) as compared to non-HIV infected patients (14/114 or 12.28%) [p = 0.41].

## Discussion

Nontuberculous mycobacteria are a recognized cause of mycobacterial infection worldwide [11]–[13]. This is also true in Africa, where TB is endemic [14]–[16]. Currently, very little data exists on the NTM species distribution in pulmonary specimens in Africa. Yet, it is well known that important differences in species distribution exist between countries and regions [17]–[18]. Though MAC has received attention due to its frequent occurrence in patients with HIV, this and other NTM have been reported also in patients with other immune suppressive diseases, in apparently normal hosts [13], [19] and smoking related chronic obstructive pulmonary disease.

The presence of NTM in the patients with chronic TB had a substantial impact on clinical management. Incorrect diagnosis of pulmonary TB due to the presence of NTM led to inappropriate or unnecessary treatment of patients with NTM isolates.

In developing countries, the presumption is that most pulmonary symptoms resembling mycobacterial disease are caused by *M. tuberculosis*. This largely stems from the lack of appropriate diagnostics in resource-limited environments as well as the endemic nature of *M. tuberculosis* in these areas [20]–[21].

Among the chronic TB cases, NTM infection was seen in 11/61 (18%) and all were receiving or were clinically empiric candidates for MDR-TB treatment. Not only is this treatment ineffective, but also expensive, often causing economic hardship, which can lead to erratic treatment and the likelihood of increased resistance.

Persons infected with *M. tuberculosis* were in some instances coinfected with NTM and few data are available on the significance of such co-infections. The NTM were found with either *M. africanum* or *M. tuberculosis* (sensu stricto) disease and were most likely colonizers. Though it is reasonable to assume that the patient's symptoms were largely due to *M. tuberculosis*, it is unclear whether the co-infecting NTM played a role in disease pathogenesis, affected time to sputum clearance or contributed to a more chronic course of the infection. NTM are found more easily in previously damaged or diseased tissue and that could explain why they were isolated from patients with preexisting TB. *M. avium* is a well-defined cause of infection and disease in both immunocompromised and otherwise normal individuals. However the role of *M. avium* in the three patients (1, 2 and 5, Table 1) who were coinfected with *M. tuberculosis* is unclear. One (Patient 5) was HIV positive as well and a pathogenic role of the co-infection cannot be discounted.

The other NTM isolated have also been associated with disease in humans. For example, members of the *M. moriokaense* group have been associated with chronic pulmonary infection [22]. *M. kumamotonense* first identified in 2006 has been implicated in clinical disease as well [23]. *M. fortuitum* has been implicated in subcutaneous and prosthetic infections and is a rare cause of pulmonary disease [24]–[27] except in those with chronic vomiting, esophageal disease [6] and immunosuppression. In normal host or those with underlying pulmonary infections, it is mainly considered to be a colonizer [11], [26], [28]. *M. kubicae* (first identified in 2000) and *M. gordonae* are possible contaminants [29]. *M. simiae*, though a reported cause of disease is generally thought to be a contaminant or colonizer of previously damaged tissue [29]–[30]. Two Patients (10 and 14, Table 1), however, met the ATS criteria for NTM disease with *M. simiae*.

*M. palustre* was first reported in 2002, and though generally isolated from environmental sources like fresh water streams and veterinary samples, it has been reported as a cause of wound infections, bacteremia [31]–[32] and lymphadenitis in humans [33] Patient 15 presented with pulmonary infection with *M. palustre*, which is a unique presentation reported for *M. palustre*. He was in the chronic disease group and was receiving a TB re-treatment regimen.

Only 22/61 (36%) patients in the chronic disease group actually had MDR disease. The rest had either susceptible disease (6 or 9.83%), single antibiotic resistance (10 or 16.40%) or NTM disease (11 or 18.03%). Without the appropriate diagnostic testing, a minimum of 27[6+10+11]/61 (44%) patients in the chronic group would have received inappropriate treatment. Twelve patients (19.67%) were culture negative. It was not possible to conclude if these culture negative patients had nonviable organisms or if they were infected by mycobacteria that did not grow on conventional media [34]–[36]. In either case these patients should likely not have been considered candidates for treatment for MDR disease. Including these 12 additional patients suggests that 39/61 (63%) patients from the chronic disease group would have been receiving an inappropriate regimen or unnecessary treatment. Most of the 18% NTM cases in the chronic group did not receive treatment for their NTM disease, and failure to identify and treat this etiology may lead to chronic, disabling symptoms and profound respiratory impairment.

Our study has some limitations that could have an impact on the results. We did not have initial laboratory data on the chronic patients preventing us from ascertaining if they had been infected only by an NTM or if they initially have had a coinfection with *M. tuberculosis*. Also, lack of follow up limits the ability to ascertain outcomes for patients especially with NTM disease. Finally the true prevalence of NTM can only be gauged during a larger epidemiological study.

Despite these limitations in our study cohort, 18% of clinically chronic TB cases could be attributed to NTM with a predominance of *M. avium*. This suggests the need to consider NTM disease in patients who fail first line and re-treatment regimens. It also highlights the need to study NTM infections in TB endemic areas through a larger cohort and to evaluate the impact of these infections on TB disease. Multidrug resistance may erroneously be suspected in these patients, and the presence of NTM in diagnostic specimens can delay the diagnosis of pulmonary malignancies in others. The significance of finding NTM should be thoroughly evaluated before treatment is started by applying the ATS criteria of NTM lung disease, because when sputum is negative and bronchial washings are positive, the odds of isolating NTM rise. Thus, careful and repeated bacteriological examination as well as clear communication between physician and microbiologist is crucial.

Lack of access to diagnostic testing is a key barrier to understanding these issues and achieving optimal outcomes. However, the cost of providing these resources is nominal, when considering the burden of inappropriate treatment. Susceptibility testing can cost between 150–200 USD in certain areas for a basic panel but is not readily available in many resource-limited countries. If suboptimal management of patients who fail treatment for TB is not acceptable in developed countries, why should the standards be different for developing countries? Increasingly, attention to the development of resistant strains of *M. tuberculosis* is a matter of urgency to prevent inappropriate use of antibiotics in the effort to achieve global TB control. This will only be possible if clinicians have the necessary diagnostic information to make correct treatment choices and if national treatment programs have access to data required to design appropriate programs based on their country needs.

In conclusion, our data suggest that NTM infections may play an important role in causing lung disease and impact the management of TB in TB-endemic environments by leading to misdiagnosis and inappropriate treatment of MDR cases particularly the clinically “chronic cases”. This highlights the necessity to consider these organisms when treating patients with putative TB treatment failures.
